# Healthcare Workers’ Knowledge, Attitudes, and Practices on Catheter-Associated Urinary Tract Infection Prevention in a Tertiary General Hospital: A Cross-Sectional Study

**DOI:** 10.7759/cureus.106902

**Published:** 2026-04-12

**Authors:** Dimitroula Giankoula, Pavlos Myrianthefs, Sofia Zyga, Georgia Kourlaba

**Affiliations:** 1 Department of Nursing, University of the Peloponnese, Tripolis, GRC; 2 Department of Nursing, National and Kapodistrian University of Athens (NKUA), Athens, GRC

**Keywords:** attitudes, catheter-associated urinary tract infection, greece, healthcare-associated infection, infection prevention, knowledge, practices

## Abstract

Background

Catheter-associated urinary tract infections (CAUTIs) remain among the most common healthcare-associated infections (HAIs) and are largely preventable through adherence to evidence-based infection prevention measures. Healthcare workers’ (HCWs) knowledge, attitudes, and practices (KAP) play a critical role in the effective implementation of these strategies. However, there is no evidence regarding HCWs’ KAP on CAUTI prevention in Greek healthcare settings. This study aimed to evaluate HCWs’ knowledge, attitudes, and practices related to CAUTI prevention in a tertiary care hospital in Greece.

Methods

An analytical cross-sectional study was conducted between September 2023 and September 2024 at a tertiary hospital in Athens. Nurses, nursing assistants, and physicians working in departments with high urinary catheter utilization were invited to participate. Data were collected using a structured questionnaire developed in accordance with international CAUTI prevention guidelines. Descriptive statistics were calculated, and non-parametric test methods were employed. The Mann-Whitney U test was used for two-group comparisons, the Kruskal-Wallis test was used for comparisons across multiple groups, and Spearman’s rank correlation coefficient was applied to examine associations with continuous variables. Logistic regression analyses were performed to identify factors associated with selected preventive practices.

Results

A total of 260 HCWs participated (response rate: 72.8%). Overall knowledge levels were moderate to high (mean score: 20.7 ± 2.1 out of 25). While most participants correctly identified key preventive measures, notable misconceptions were observed regarding prophylactic antimicrobial use after catheter insertion and appropriate catheter fixation techniques. Attitudes toward CAUTI prevention were generally positive, with the majority recognizing the importance of education and appropriate catheter management. Despite the high level of knowledge, a clear knowledge-practice gap was identified. Although the participants correctly identified key preventive measures, adherence to routine maintenance practices was markedly lower, particularly for hand hygiene before catheter manipulation (26.2%) and the maintenance of a continuously closed drainage system (66.5%). Greater professional experience (adjusted odds ratio {aOR}, 2.22; 95% confidence interval {CI}, 1.08-4.56) and higher knowledge scores (aOR, 1.18 per point; 95% CI, 1.02-1.37) were independently associated with correct catheter fixation. Additionally, positive perceptions regarding the role of catheter management in reducing morbidity were strongly associated with maintaining a closed drainage system (aOR, 6.65; 95% CI, 1.72-25.66).

Conclusions

Although HCWs demonstrated adequate knowledge and favorable attitudes toward CAUTI prevention, important gaps remain in the consistent implementation of recommended practices. Targeted educational initiatives and organizational strategies focusing on catheter maintenance and adherence to prevention guidelines may improve clinical practice and reduce CAUTI risk.

## Introduction

According to the most recent point prevalence survey conducted by the European Centre for Disease Prevention and Control (ECDC), urinary tract infections (UTIs) were the second most frequently reported healthcare-associated infection (HAI), accounting for 19.2% of all reported cases. Importantly, 61.9% of HAIs classified as UTIs were associated with the presence of an indwelling urinary catheter [1]. Consistent with these findings, catheter-associated urinary tract infections (CAUTIs) are widely recognized as a major contributor to the HAI burden internationally, driven largely by increasing antimicrobial resistance, particularly among common pathogens such as *Escherichia coli* and *Klebsiella pneumoniae*. Improving diagnostic speed, standardizing data reporting, and promoting more targeted antibiotic use are essential steps to reduce ineffective treatments and improve patient outcomes [2].

In Greece, surveillance data on CAUTIs remain limited. In 2010, a study conducted across three intensive care units (ICUs) in Athens identified CAUTIs as the third most common type of HAIs, with an incidence rate of 4.2 per 1,000 catheter-days [3]. A subsequent study in a similar ICU setting reported comparable findings, ranking CAUTIs as the third most frequent infection, with a pooled incidence rate of 3.5 per 1,000 catheter-days [4].

The occurrence of CAUTI is strongly influenced by factors, including the duration of catheter use, the technique of catheterization, and the patient’s existing health conditions [5]. Urinary catheters are widely used in the emergency department and hospital wards in general, with 12%-16% of adults using them at least once during their hospitalization, which increases the risk of CAUTI by 3%-7% daily [6]. Complications arising from CAUTIs contribute to patient morbidity, prolonged hospitalization, increased healthcare costs, and elevated mortality rates [7]. Epidemiological estimates indicate that UTIs are associated with more than 13,000 deaths annually [8].

CAUTIs are largely preventable through adherence to evidence-based infection prevention and control measures, including appropriate indications for catheter insertion, strict aseptic technique, the maintenance of a closed drainage system, and daily reassessment with prompt removal when no longer indicated [9]. Despite the availability of clear guidelines, gaps between recommended practices and routine clinical care remain common [10].

Healthcare workers’ (HCWs) knowledge, attitudes, and practices (KAP) play a central role in the implementation of infection prevention measures and have been linked to HAI prevention performance [11]. Assessing HCWs’ KAP and identifying factors associated with suboptimal preventive behaviors can inform targeted interventions and support local HAI prevention programs [12].

Despite the extensive documentation of antimicrobial resistance and HAIs in Greece by the ECDC through the 2016-2017 and 2022-2023 point prevalence surveys, studies focusing specifically on HCWs’ KAP regarding CAUTI prevention in Greek hospitals are lacking [1,13,14]. Therefore, this analytical cross-sectional study, in a tertiary care hospital in Greece, had two objectives: (1) to assess healthcare workers’ knowledge, attitudes, and practices (KAP) regarding catheter-associated urinary tract infection prevention and (2) to identify demographic, professional, knowledge-related, and perception-related factors associated with selected preventive practices.

## Materials and methods

Study design and participants

An analytical cross-sectional survey was conducted at the General Hospital of Athens, Evangelismos, between September 2023 and September 2024. The eligible population consisted of healthcare workers from nine departments with a high utilization of indwelling urinary catheters. Specifically, nurses, nursing assistants, and physicians working in four internal medicine wards, two intensive care units, one oncology department, one cardiac intensive care unit (CICU), and one interdepartmental nursing department were invited to participate.

Clinical interns, visiting trainees from other institutions, outpatient staff, and medical technicians not directly involved in patient care were excluded.

Sample size

A total-population (total enumeration) approach was used in the selected departments, in which all eligible healthcare workers were invited to participate. However, because participation was voluntary, the final sample may be subject to response-related selection bias.

The sample size for the questionnaire survey was determined based on a cross-sectional design using the following calculation formula: n = (Z²α/2 × p × q) / d².

To determine the appropriate sample size for the survey, the following parameters were used: a confidence level of 99% (corresponding to a Zₐ/2 value of 2.576), an expected proportion (p) of 0.65 representing the anticipated rate of prevention practice, and a margin of error set at 10% of p. Accordingly, q was defined as 1 - p. A t-sided test was employed in the calculation. Using a finite-population correction for the study population of 357 individuals, the minimum required sample size was determined. To account for potential non-response and incomplete questionnaires, a 20% increase was added to the calculated sample size. Based on these criteria, the final target sample size was set at 215 participants.

A total of 260 healthcare workers participated (response rate: 72.8%). Because the questionnaire was anonymous, no data were available for non-responders, and comparisons between responders and non-responders could not be performed.

Data collection

To serve the objective of the present study, a questionnaire was developed from scratch based on the latest guidelines (Strategies to prevent catheter-associated urinary tract infections in acute-care hospitals: 2022 Update) issued by SHEA/IDSA/APIC, as well as international CAUTI prevention and control guidelines and relevant literature [6,9,15,16]. Content validity was assessed by three experts with experience in infection prevention and catheter care, who reviewed the questionnaire for the relevance and appropriateness of the included items. Face validity was evaluated by a group of healthcare professionals (four nurses and two physicians) to ensure the clarity and comprehensibility of the questions.

The questionnaire was structured into four sections. The first section collected demographic information, including sex, age, education, years of professional experience, specialization (physician, nurse, or nurse assistant), current hospital department, and prior training in infection control, either general or specific to CAUTI prevention within the preceding 12 months. The second section assessed knowledge and was subdivided into two domains: (i) basic knowledge of Foley catheter indications, evaluated through 10 items, and (ii) knowledge of CAUTI prevention and control measurements, assessed through 15 items. Responses were recorded as true, false, or I do not know. The third section evaluated attitudes toward CAUTI prevention and perceived burden using 12 items rated on a five-point Likert scale. The fourth section examined self-reported practices, comprising five dichotomous (yes/no) items focused on infection prevention behaviors implemented in routine clinical care. Together, these sections provided a comprehensive assessment of HCWs’ knowledge, attitudes, and practices regarding CAUTI prevention, thereby supporting the questionnaire’s relevance to clinical settings. The survey was administered in paper format by a member of the hospital’s infection control committee. Throughout the study, all data were kept confidential.

Data analysis

Descriptive statistics were computed for all study variables, including demographic characteristics, knowledge items, attitude items, and practice-related outcomes. Categorical variables were presented as counts and percentages, while continuous variables were reported as means with standard deviations (SD) or medians with interquartile ranges (IQR), depending on their distribution; normality was assessed through the visual inspection of histograms and Q-Q plots. Knowledge items were scored dichotomously (1 = correct; 0 = incorrect or “I do not know”). A total knowledge score was calculated for each participant by summing the number of correct responses. The internal consistency of the knowledge section (overall and by sub-domains) was assessed using the Kuder-Richardson coefficient (KR-20). Because knowledge scores were not normally distributed, associations with demographic and professional variables were examined using non-parametric tests. Mann-Whitney U tests were applied for two-group comparisons, Kruskal-Wallis tests for variables with more than two categories, and Spearman’s rank correlation for continuous predictors. Perception items were analyzed individually and were not combined into a composite score, as they reflected conceptually distinct aspects of participants’ perceptions rather than a single underlying dimension.

Separate multivariable logistic regression models were fitted for selected CAUTI prevention practices. Each outcome was defined as binary (yes/no) based on self-reported adherence and was modeled separately as a dependent variable in individual logistic regression analyses. The outcomes included (1) correct catheter fixation after insertion, (2) the maintenance of a continuously closed drainage system, (3) the use of personal protective equipment (PPE) due to the risk of exposure to biological fluids, (4) the recording of catheter removal date, and (5) appropriate technique for the removal of the fixation medium. These outcomes were selected based on their clinical relevance and observed variability in adherence. Univariable logistic regression analyses were first performed to estimate unadjusted odds ratios (ORs) with 95% confidence intervals (CIs). Variables that were statistically significant in univariable analyses were subsequently included in the corresponding multivariable models, following a data-driven approach to adjustment for potential confounding. Formal assessment of effect modification (interaction) was not performed, given the exploratory nature of the analyses. Adjusted ORs with 95% CIs were reported. A two-tailed p-value of <0.05 was considered statistically significant. All analyses were performed using the Stata software (StataCorp LLC, College Station, TX).

Ethical considerations

The study received ethical approval from the Research Ethics and Deontology Committee of Evangelismos Hospital (approval number: 326). Furthermore, the participants were informed in the questionnaire’s introductory note about the study’s purpose, and, if they wished, they could proceed to complete it. The questionnaire was anonymous, and participation in the study was completely voluntary. There were no potential risks to the participants. The information collected is solely for this research and will be kept strictly confidential. This research was conducted in compliance with the principles of the Declaration of Helsinki.

## Results

Table 1 presents the demographic and professional profiles of the participants. Most were women (n = 199, 76.5%) with a mean age of 40.1 years (SD: 9.8), and registered nurses formed the largest group (n = 111, 42.7%), followed by nursing assistants (n = 56, 21.5%), medical specialists (n = 55, 21.2%), and medical residents (n = 38, 14.6%). Work experience was substantial overall (median, 15 years; IQR, 6-23). Only 59 participants (22.7%) reported previous education in infection control, while 85 participants (32.7%) had received CAUTI prevention training within the previous 12 months.

**Table 1:** Demographic characteristics of healthcare professionals (n = 260) Data are presented as n (%) for categorical variables and mean ± standard deviation (SD) or median (interquartile range, IQR) for continuous variables CAUTI: catheter-associated urinary tract infection

Demographics	N (%)
Total sample (n = 260)	
Gender	
Female	199 (76.5)
Male	61 (23.5)
Age	
Mean ± SD	40.1 ± 9.8
Age group (years)	
20-35	87 (33.5)
35-50	119 (45.8)
≥50	54 (20.8)
Education	
Public/private school	54 (20.8)
Bachelor’s degree	94 (36.2)
Master of Science	87 (33.5)
PhD	21 (8.1)
Postdoc	4 (1.5)
Professional status	
Nursing assistant	56 (21.5)
Registered nurse	111 (42.7)
Medical resident	38 (14.6)
Medical specialist	55 (21.2)
Work experience in the current department	
Median (P25-P75)	5 (2-11.5)
Years of experience in the current department (categorical)	
<5	128 (49.2)
5, 10	48 (18.5)
≥10	84 (32.3)
Work experience in general	
Median (P25-P75)	15 (6-23)
Years of experience in general (categorical)	
<10	98 (37.7)
10, 20	68 (26.2)
≥20	94 (36.2)
Previous education in infection control in general	
Yes	59 (22.7)
No	201 (77.3)
Previous education in CAUTI in the last 12 months	
Yes	85 (32.7)
No	175 (67.3)

Assessment of knowledge

Table 2 presents the level of knowledge regarding the correct indications for urinary catheter placement and preventive measures for CAUTIs. Knowledge regarding appropriate indications for Foley placement was generally moderate to high. Correct responses exceeded 70% for most items. However, lower levels of correct responses were observed for catheter use in the context of palliative care in terminal patients (n = 143, 55.0%) and prolonged immobilization (n = 162, 62.3%), indicating areas of uncertainty. The mean score for indications was 7.3 (SD: 1.5) out of 10, with a median of 8 (IQR: 6-8). The overall knowledge of prevention measures was high. Nearly all participants correctly identified essential practices. However, notable misconceptions were identified. One hundred eighty-seven participants (71.9%) correctly recognized that routine bladder flushing with antimicrobial or iodine solutions does not prevent CAUTI, and 151 (58.3%) incorrectly believed that prophylactic antimicrobial therapy should be administered after catheter insertion. Additionally, knowledge regarding proper catheter fixation was limited, with only 88 participants (34.1%) answering correctly. The mean prevention score was 13.3 (SD: 1.2) out of 15, with a median of 14 (IQR: 13-14). The overall knowledge score (range: 0-25) showed a mean of 20.7 (SD: 2.1) and a median of 21 (IQR: 19-22). The internal consistency of the total knowledge scale was acceptable (Kuder-Richardson = 0.52).

**Table 2:** Percentage of participants providing the correct answer at each knowledge item Data are presented as numbers (n) and percentages (%). Knowledge scores are reported as mean ± standard deviation (SD) and median (interquartile range, IQR). The internal consistency of the knowledge domains was assessed using the Kuder-Richardson coefficient (KR-20)

Questions	N (%)
A. Indications for urinary catheter placement	
1. Narrowing of the urethra obstructing urine flow: indicated	184 (70.8)
2. Neurogenic bladder due to paraplegia or tetraplegia: indicated	197 (75.8)
3. Volume monitoring of urine output in an ambulatory patient without a pathological problem from the urinary tract: not indicated	218 (83.8)
4. Collection of urine samples for culture and antibiotic susceptibility testing of microorganisms (C&S) in ambulatory patients: not indicated	229 (88.1)
5. In patients who are expected to receive a large volume of intravenous fluids or diuretics during surgery: indicated	231 (88.8)
6. In patients with prolonged immobilization: indicated	162 (62.3)
7. To aid the healing of pressure ulcers in female incontinent patients: indicated	181 (69.6)
8. In the context of palliative care in a terminal patient: indicated	143 (55.0)
9. As a substitute for nursing care in patients with incontinence: not indicated	168 (64.6)
10. Before any kind of surgery as a routine procedure: not indicated	197 (75.8)
n	260
Mean ± SD	7.3 ± 1.5
Median (p25-p75)	8 (6-8)
Kuder-Richardson	0.49
B. Prevention measures	
1. Hand hygiene should be applied immediately before and after any handling of the urinary catheter	259 (99.6)
2. An appropriately sized urinary catheter should be used	260 (100)
3. The urinary catheter should be inserted only when necessary and removed as soon as possible to prevent urinary tract infection associated with urinary catheter use	258 (99.2)
4. Other methods of urinary drainage should be considered before urinary catheter placement	238 (92.2)
5. The urinary catheter should only be inserted by personnel trained in urinary catheter insertion under aseptic conditions	250 (96.2)
6. Crushing of the urinary catheter should be avoided to keep the flow of urine unobstructed	255 (98.1)
7. Flushing the bladder with an antimicrobial or iodine solution at least once a day will prevent urinary tract infection associated with urinary catheter use	187 (71.9)
8. The urine collector should be emptied regularly to avoid overflow of urine	251 (96.9)
9. The urinary collector should always be kept below the level of the bladder	259 (99.6)
10. The urine collector should not rest on the floor	247 (95.0)
11. Prophylactic antimicrobial treatment should be given after the insertion of the urinary catheter	151 (58.3)
12. The urinary catheter should be removed as soon as the patient does not need it	254 (97.7)
13. The urinary catheter should be clipped on the inner side of the thigh in women or on the lower abdomen in men	88 (34.1)
14. Sterile lubricants should be used when inserting the urinary catheter to reduce the irritation of the urethra	241 (93.1)
15. The perineal area in women should be cleaned from front to back	249 (95.8)
Total score for prevention measures (0-15)	
n	253
Mean ± SD	13.3 ± 1.2
Median (p25-p75)	14 (13-14)
Kuder-Richardson	0.31
Total knowledge score (0-25)	
n	253
Mean (SD)	20.7 (2.1)
Median (p25-p75)	21 (19-22)
Kuder-Richardson	0.52

In Table 3, the associations of knowledge with demographic and professional variables were examined. Overall, median knowledge scores were high across most sociodemographic and professional subgroups. The participants who reported general previous education in infection control demonstrated significantly higher knowledge scores on indications for urinary catheter placement (median: 8 {7-9} versus 7 {6-8}, p = 0.009). However, this difference was not observed in the knowledge score on prevention measurements (p = 0.337). In contrast, previous education specifically on CAUTI within the last 12 months was significantly associated with higher knowledge scores on the prevention measurements (median: 14 {13-14} versus 13 {13-14}, p = 0.033) but not on the knowledge score on indications for urinary catheter placement (p = 0.891).

**Table 3:** Association of knowledge score with demographics Continuous variables are presented as median (p25-p75). Comparisons between two groups were performed using the Mann-Whitney (Wilcoxon rank-sum) test. Comparisons among more than two groups were performed using the Kruskal-Wallis test. Correlations were assessed using Spearman’s rank correlation coefficient (rho). Test statistics (z, χ², and rho) are reported with corresponding p-values. Statistical significance was defined as p < 0.05 CAUTI: catheter-associated urinary tract infection

	Knowledge score on indications for urinary catheter placement (0-10)				Knowledge score on indications for urinary catheter placement (0-15)			
	n	Median (p25-p75)	Test statistic	P-value	n	Median (p25-p75)	Test statistic	P-value
Gender								
Female	199	8 (6-8)	z = 1.491	0.136	194	14 (13-14)	z = 1.235	0.217
Male	61	7 (6-8)			59	13 (12-14)		
Age								
Spearman’s	260	Rho = 0.023		0.715	253	0.093		0.139
Age group (years)								
20-35	87	7 (6-9)	χ²(2) = 0.181	0.913	86	13 (12-14)	χ²(2) = 4.557	0.102
35, 50	119	8 (6-8)			115	14 (13-14)		
*≥*50	54	7.5 (6-8)			52	13 (13-14)		
Education								
Public/private school	54	8 (7-8)	χ²(2) = 0.619	0.892	53	14 (13-14)	χ²(2) = 2.899	0.407
Bachelor’s degree	94	8 (6-9)			91	14 (13-14)		
Master of Science	87	7 (6-8)			85	13 (13-14)		
PhD/postdoc	25	7 (6-8)			24	14 (13-14.5)		
Professional status								
Nursing assistant	56	8 (7-8)	χ²(2) = 2.835	0.418	55	14 (13-14)	χ²(2) = 6.337	0.096
Registered nurse	111	8 (6-9)			106	13 (13-14)		
Medical resident	38	7.5 (6-8)			38	13.5 (12-14)		
Medical specialist	55	7 (6-8)			54	14 (13-14)		
Work experience in the current department								
Spearman’s	260	Rho = 0.048		0.441	253	0.106		0.092
Categorical (years of work experience in the current department)								
<5	128	8 (6-8)	χ²(2) = 1.609	0.447	123	13 (12-14)	χ²(2) = 4.989	0.082
5-10	48	7 (6-8)			47	14 (13-14)		
≥10	84	8 (6-8)			83	14 (13-14)		
Work experience in general								
Spearman’s	260	Rho = 0.047		0.453	253	0.116		0.064
Categorical (years of work experience in general)								
<10	98	7 (6-8)	χ²(2) = 2.033	0.362	95	13 (12-14)	χ²(2) = 5.198	0.074
10-20	68	8 (6.5-9)			67	14 (13-14)		
≥20	94	8 (6-8)			91	14 (13-14)		
Previous education in infection control in general								
Yes	59	8 (7-9)	z = 2.594	0.009	57	13 (12-14)	z = -0.960	0.337
No	201	7 (6-8)			196	14 (13-14)		
Previous education in CAUTI in the last 12 months								
Yes	85	8 (6-8)	z = -0.138	0.891	82	14 (13-14)	z = 2.132	0.033
No	175	8 (6-8)			171	13 (13-14)		

Attitude toward CAUTI

Table 4 presents the participants’ perceptions of CAUTI prevention. Overall, the respondents demonstrated generally positive perceptions. One hundred sixty-two participants (62.3%) agreed/strongly agreed that CAUTIs are a serious condition that may lead to death. Nearly all respondents (n = 252, 96.9%) agreed or strongly agreed that education helps prevent CAUTI, and 244 participants (94.2%) agreed that the proper placement and maintenance of urinary catheters reduce CAUTI-related morbidity. In contrast, perceptions were more divided regarding routine catheter replacement as a preventive measure: only 114 (43.9%) agreed/strongly agreed that routine catheter changes help prevent CAUTI. Workload was identified as a potential barrier following the guideline implementations. Approximately 69 participants (26.6%) agreed that workload affects their ability to implement CAUTI prevention guidelines.

**Table 4:** Participants’ perception of CAUTI prevention Responses were measured using a five-point Likert scale and are presented as number (n) and percentage (%). Totals may vary due to missing values CAUTI: catheter-associated urinary tract infection

Questions	Disagree/strongly disagree	Neither agree nor disagree	Agree/strongly agree
	n (%)	n (%)	n (%)
1. Urinary tract infection associated with urinary catheter use is a serious disease that can lead to death	29 (11.2)	69 (26.5)	162 (62.3)
2. Changing urinary catheters in routine is a practice that helps prevent urinary tract infections associated with urinary catheter use	110 (42.3)	36 (13.8)	114 (43.9)
3. The occurrence of urinary tract infection associated with urinary catheter use cannot be avoided in patients who have undergone bladder catheterization	161 (62)	49 (18.8)	50 (19.2)
4. Education on the basics of urinary catheter care helps prevent urinary tract infections associated with urinary catheter use	0 (0)	8 (3.1)	252 (96.9)
5. Healthcare professionals can remove the catheter whenever it is convenient for them	206 (79.5)	39 (15.1)	14 (5.4)
6. Urinary tract infections associated with urinary catheter use are a common problem	10 (3.8)	31 (11.9)	219 (84.2)
7. Aseptic technique required for the removal of the Foley urinary catheter	88 (33.9)	32 (12.4)	139 (53.7)
8. Workload affects my ability to implement the guidelines for the prevention of CAUTI	139 (53.7)	51 (19.7)	69 (26.6)
9. The proper placement and maintenance of the urinary catheter can reduce the risk of CAUTI-related morbidity	2 (0.8)	13 (5)	244 (94.2)
10. The proper placement and maintenance of the urinary catheter can reduce the risk of CAUTI-related mortality	4 (1.6)	29 (11.3)	223 (87.1)
11. In the event that I observed improper practices being followed during urinary catheter insertion, I would intervene and politely point out the error	3 (1.2)	14 (5.4)	242 (93.4)
12. Urinary catheter dwell time contributes significantly to the occurrence of CAUTI	2 (0.8)	17 (6.6)	240 (92.6)

Practice on the prevention of CAUTI

Table 5 presents participants’ self-reported practices for CAUTI prevention. Overall adherence to recommended practices was high for several key infection prevention measures, but notable gaps were observed in aspects of catheter care. A total of 251 participants (96.5%) reported performing hand hygiene before and after urinary catheter insertion. However, substantially lower proportions reported performing hand hygiene during routine catheter care, with only 68 (26.2%) reporting hand hygiene before handling the catheter and 46 (17.7%) after insertion as separate practices. These items reflected different procedural contexts rather than mutually exclusive responses, indicating stronger adherence during catheter insertion compared to routine maintenance. Two hundred forty-eight participants (95.4%) reported using sterile equipment such as sterile gloves and gauze, and 241 (92.7%) reported applying aseptic technique. Two hundred fifteen participants (82.7%) reported cleaning the urethral orifice with an antiseptic solution before insertion, and 197 (75.8%) ensured proper catheter fixation after insertion. Two hundred twenty-seven responders (87.3%) reported using single-use lubricating gel. Of the HCWs, 243 (93.5%) reported keeping the catheter and tubing free of folds to ensure unobstructed urine flow, and 251 (96.5%) maintained the urinary drainage system below bladder level. Two hundred thirty-one (88.8%) stated that they regularly emptied the urine collection bag and used a separate container for each patient. However, maintaining a continuously closed drainage system showed lower adherence, with only 173 of the participants (66.5%) reporting compliance. The use of appropriate personal protective equipment (PPE) during the routine handling of the catheter was reported by 235 participants (90.4%). Two hundred six of the responders (79.2%) used appropriate personal protective measures due to the risk of exposure to biological fluids. Two hundred fifty-five (98.1%) used a 10 mL or 20 mL syringe to deflate the catheter balloon appropriately. Two hundred two participants (77.7%) reported recording the date of catheter removal, while 195 participants (75.0%) used the correct technique to remove the fixation medium.

**Table 5:** Percentage of self-reported practices Data are presented as numbers (n) and percentages (%)

Questions	n	%
1. When do you practice hand hygiene?		
1.1. Before and after the insertion of the urinary catheter	251	96.5
1.2. Before handling the urinary catheter	68	26.2
1.3. After the insertion of the urinary catheter	46	17.7
2. Which of the following do you use/wear when inserting the urinary catheter?		
2.1. Sterile equipment, such as sterile gloves and gauze	248	95.4
2.2. Aseptic technique	241	92.7
2.3. Cleaning the urethral orifice with an antiseptic solution	215	82.7
2.4. Ensure the correct method of fixation of the urinary catheter after insertion	197	75.8
2.5. Lubricating gel in a single package	227	87.3
3. Which of the following do you use/maintain during the routine care of the urinary catheter?		
3.1. Keeping the urinary catheter and urinary collector tube free of folds for unobstructed flow of urine	243	93.5
3.2. Keeping the urinary tract at a level lower than the bladder	251	96.5
3.3. Emptying the urine collector regularly and using a different urine collection container for each patient	231	88.8
3.4. Keeping the system continuously closed	173	66.5
3.5. Use of appropriate personal protective equipment (PPE) according to the guidelines when handling the urinary catheter	235	90.4
4. Which of the following do you use/wear when removing the urinary catheter?		
4.1. Use of PPE, due to the risk of exposure to biological fluids	206	79.2
4.2. Use a 10 or 20 mL syringe to remove water from the urinary catheter stabilization balloon	255	98.1
4.3. Recording the date of the removal of the urinary catheter	202	77.7
4.4. Use of the appropriate technique to remove the fixation medium	195	75.0

Factors associated with CAUTI prevention practices (multivariable analysis)

A multivariable logistic regression analysis was applied to examine whether experience and perception-related factors were associated with adherence to specific behaviors. However, most sociodemographic characteristics were not consistently associated with outcomes.

In Table 6, ensuring the correct method of catheter fixation after insertion was analyzed. The participants with ≥20 years of general work experience had higher odds of correct fixation compared with those with <10 years (adjusted odds ratio {aOR}, 2.22; 95% CI, 1.08-4.56; p = 0.030). In addition, the total knowledge score was positively associated with correct fixation, with each one-point increase associated with an 18% increase in odds (aOR, 1.18 per point; 95% CI, 1.02-1.37; p = 0.026).

**Table 6:** Multivariable logistic regression analysis for the outcome “ensuring correct method of fixation of the urinary catheter after insertion” (yes versus no) Data are presented as odds ratios (OR) with 95% confidence intervals (CI). Adjusted odds ratios (aOR) were obtained from multivariable logistic regression analysis. Statistical significance was defined as p < 0.05 *Statistically significant

Predictor	Unadjusted OR (95% CI)	P-value	Adjusted OR (95% CI)	P-value
Years of work experience in general (reference: <10 years)				
10, 20	1.71 (0.84-3.49)	0.138	1.64 (0.77-3.49)	0.197
*≥*20	2.36 (1.19-4.68)	0.014*	2.22 (1.08-4.56)	0.030*
Total knowledge score (1 point)	1.23 (1.07-1.42)	0.004*	1.18 (1.02-1.37)	0.026*

In Table 7, maintaining a continuously closed drainage system was associated with gender, profession, experience, and perceptions. Men had higher odds of maintaining a closed system compared with women (aOR, 2.60; 95% CI, 1.13-6.02; p = 0.025). Compared with registered nurses, nursing assistants (aOR, 0.08; 95% CI, 0.01-0.84; p = 0.035) and medical residents (aOR, 0.29; 95% CI, 0.11-0.73; p = 0.009) were less likely to report this practice. The participants with 10-20 years (aOR, 3.26; 95% CI, 1.36-7.82; p = 0.008) and ≥20 years (aOR, 2.31; 95% CI, 1.08-4.97; p = 0.032) of general work experience had higher odds of maintaining a closed system compared with those with <10 years. Agreeing that proper catheter placement and maintenance reduce CAUTI-related morbidity (perception question 9) was strongly associated with maintaining a closed system (aOR, 6.65; 95% CI, 1.72-25.66; p = 0.006).

**Table 7:** Multivariable logistic regression analysis for the outcome “maintaining the system continuously closed” (yes versus no) Data are presented as odds ratios (OR) with 95% confidence intervals (CI). Adjusted odds ratios (aOR) were estimated using multivariable logistic regression analysis. Statistical significance was defined as p < 0.05 *Statistically significant

Predictors	Unadjusted OR (95% CI)	P-value	Adjusted OR (95% CI)	P-value
Gender (reference: woman)				
Man	1.94 (1.00-3.77)	0.049*	2.60 (1.13-6.02)	0.025*
Education level (reference bachelor’s)				
Public/private school	0.65 (0.32-1.30)	0.224	5.37 (0.53-54.34)	0.155
Master of Science	0.69 (0.38-1.28)	0.246	0.87 (0.42-1.77)	0.696
PhD/postdoc	5.13 (1.13-23.22)	0.034*	3.02 (0.55-16.59)	0.204
Profession (reference: registered nurse)				
Nursing assistant	0.42 (0.21-0.82)	0.012*	0.08 (0.01-0.84)	0.035*
Medical resident	0.20 (0.09-0.43)	<0.001*	0.29 (0.11-0.73)	0.009*
Medical specialist	1.52 (0.68-3.41)	0.311	0.68 (0.26-1.78)	0.436
Years of work experience in general (reference: <10 years)				
10, 20	4.41 (2.14-9.08)	<0.001*	3.26 (1.36-7.82)	0.008*
≥20	3.04 (1.65-5.59)	<0.001*	2.31 (1.08-4.97)	0.032*
Q9. The proper placement and maintenance of the urinary catheter can reduce the risk of CAUTI-related morbidity (reference: strongly disagree/disagree/neither agree nor disagree)				
Agree/strongly agree	6.20 (1.91-20.09)	0.002*	6.65 (1.72-25.66)	0.006*

In Table 8, the use of PPE during urinary catheter removal was examined. Compared with registered nurses, nursing assistants (aOR, 0.25; 95% CI, 0.11-0.61; p = 0.002) and medical residents (aOR, 0.28; 95% CI, 0.11-0.72; p = 0.008) had lower odds of PPE use. Previous CAUTI education was associated with substantially higher odds of PPE use (aOR, 2.90; 95% CI, 1.27-6.58; p = 0.011). Additionally, agreeing that routine catheter changes help prevent CAUTI (perception item 2) was associated with higher odds of PPE use (aOR, 2.27; 95% CI, 1.10-4.70; p = 0.026).

**Table 8:** Multivariable logistic regression analysis for the outcome “use of PPE due to the risk of exposure to biological fluids” (yes versus no) Data are presented as odds ratios (OR) with 95% confidence intervals (CI). Adjusted odds ratios (aOR) were estimated using multivariable logistic regression analysis. Statistical significance was defined as p < 0.05 *Statistically significant CAUTI, Catheter-associated urinary tract infection; PPE, personal protective equipment

Predictors	Unadjusted OR (95% CI)	P-value	Adjusted OR (95% CI)	P-value
Profession (reference: registered nurse)				
Nursing assistant	0.26 (0.11-0.58)	0.001*	0.25 (0.11-0.61)	0.002*
Medical resident	0.18 (0.08-0.45)	<0.001*	0.28 (0.11-0.72)	0.008*
Medical specialist	0.62 (0.24-1.57)	0.314	0.78 (0.28-2.13)	0.622
Previous education in CAUTI (reference: no)				
Yes	2.92 (1.35-6.31)	0.006*	2.90 (1.27-6.58)	0.011*
Q2. Changing urinary catheter in routine is a practice that helps prevent urinary tract infections associated with urinary catheter use (reference: strongly disagree/disagree/neither agree nor disagree)				
Agree/strongly agree	1.94 (1.02-3.66)	0.042*	2.27 (1.10-4.70)	0.026*
Q6. Urinary tract infections associated with urinary catheter use are a common problem (reference: strongly disagree/disagree/neither agree nor disagree)				
Agree/strongly agree	0.26 (0.08-0.88)	0.030*	0.30 (0.08-1.07)	0.064
Q11. If I observed improper practices being followed during urinary catheter insertion, I would intervene and politely point out the error (reference: strongly disagree/disagree/neither agree nor disagree)				
Agree/strongly agree	2.90 (1.05-8.03)	0.040*	2.46 (0.79-7.69)	0.122

In Table 9, the date of urinary catheter removal was assessed. Compared with participants with a bachelor’s degree, those with school-level education had higher odds of recording removal dates (aOR, 18.82; 95% CI, 1.34-263.82; p = 0.029), though the confidence interval was extensive, indicating an imprecise estimate likely influenced by small subgroup counts. Compared with registered nurses, nursing assistants were less likely to record removal dates (aOR, 0.04; 95% CI, 0.003-0.55; p = 0.016), also with a wide CI suggesting limited precision. Agreeing that aseptic technique is required for catheter removal (perception question 7) was positively associated with this practice (aOR, 2.39; 95% CI, 1.17-4.88; p = 0.017), as was agreeing that proper catheter placement and maintenance reduce CAUTI-related mortality (perception question 10; aOR, 6.44, 95% CI, 2.70-15.37; p < 0.001).

**Table 9:** Multivariable logistic regression analysis for the outcome “recording the date of urinary catheter removal” (yes versus no) Data are presented as odds ratios (OR) with 95% confidence intervals (CI). Adjusted odds ratios (aOR) were estimated using multivariable logistic regression analysis. Statistical significance was defined as p < 0.05 *Statistically significant CAUTI: catheter-associated urinary tract infection

Predictor	Unadjusted OR (95% CI)	P-value	Adjusted OR (95% CI)	P-value
Education level (reference: bachelor’s)				
Public/private school	0.60 (0.26-1.38)	0.228	18.82 (1.34-263.82)	0.029*
Master of Science	0.44 (0.22-0.91)	0.027*	0.37 (0.16-0.86)	0.020*
PhD/postdoc	1.00 (0.30-3.32)	0.996	0.50 (0.10-2.52)	0.404
Profession (reference: registered nurse)				
Nursing assistant	0.80 (0.39-1.66)	0.554	0.04 (0.003-0.55)	0.016*
Medical resident	0.900 (0.39-2.09)	0.806	0.77 (0.28-2.10)	0.610
Medical specialist	3.21 (1.16-8.88)	0.024*	2.08 (0.61-7.12)	0.243
Years of work experience in general (reference: <10 years)				
10, 20	2.57 (1.08-6.11)	0.033*	2.23 (0.76-6.53)	0.145
≥20	0.94 (0.50-1.80)	0.864	0.97 (0.41-2.32)	0.950
Q7. Aseptic technique required for the removal of the Foley urinary catheter (reference: strongly disagree/disagree/neither agree nor disagree)				
Agree/strongly agree	2.28 (1.25-4.15)	0.007*	2.39 (1.17-4.88)	0.017*
Q10. The proper placement and maintenance of the urinary catheter can reduce the risk of CAUTI-related mortality (reference: strongly disagree/disagree/neither agree nor disagree)				
Agree/strongly agree	6.61 (3.05-14.32)	<0.001*	6.44 (2.70-15.37)	<0.001*

In Table 10, using the appropriate technique to remove the fixation medium was evaluated. Agreeing that proper catheter placement and maintenance reduce CAUTI-related mortality (perception question 10) was associated with higher odds of correct technique (aOR, 2.81; 95% CI, 1.25-6.31; p = 0.012). Likewise, the participants who reported willingness to intervene when observing improper practices (perception question 11) had higher odds of correct technique (aOR, 3.33; 95% CI, 1.18-9.36; p = 0.023). Conversely, agreeing that CAUTIs are a common problem (perception question 6) was associated with lower odds of correct technique (aOR, 0.22; 95% CI, 0.06-0.76; p = 0.017), a finding that should be interpreted cautiously given the potential for residual confounding and response-pattern effects.

**Table 10:** Multivariable logistic regression analysis for the outcome “use of appropriate technique to remove the fixation medium” (yes versus no) Data are presented as odds ratios (OR) with 95% confidence intervals (CI). Adjusted odds ratios (aOR) were estimated using multivariable logistic regression analysis. Statistical significance was defined as p < 0.05 *Statistically significant CAUTI: catheter-associated urinary tract infection

Predictors	Unadjusted OR (95% CI)	P-value	Adjusted OR (95% CI)	P-value
Q3. The occurrence of urinary tract infection associated with urinary catheter use cannot be avoided in patients who have undergone bladder catheterization (reference: strongly disagree/disagree/neither agree nor disagree)				
Agree/strongly agree	0.51 (0.26-0.99)	0.048*	0.53 (0.26-1.07)	0.076
Q6. Urinary tract infections associated with urinary catheter use are a common problem (reference: strongly disagree/disagree/neither agree nor disagree)				
Agree/strongly agree	0.20 (0.06-0.67)	0.009*	0.22 (0.06-0.76)	0.017*
Q10. The proper placement and maintenance of the urinary catheter can reduce the risk of CAUTI-related mortality (reference: strongly disagree/disagree/neither agree nor disagree)				
Agree/strongly agree	2.48 (1.16-5.30)	0.019*	2.81 (1.25-6.31)	0.012*
Q11. In the event that I observed improper practices being followed during urinary catheter insertion, I would intervene and politely point out the error (reference: strongly disagree/disagree/neither agree nor disagree)				
Agree/strongly agree	3.74 (1.38-10.14)	0.010*	3.33 (1.18-9.36)	0.023*

## Discussion

CAUTI remains one of the most frequent HAIs, and device use is a key driver of preventable risk. In the European acute-care hospital point prevalence survey (2022-2023), urinary tract infections represented ~19% of HAIs, and among patients with a UTI, a large proportion had recent urinary catheter exposure, highlighting the ongoing relevance of catheter stewardship and safe maintenance practices in routine care [1].

In our study, overall knowledge regarding urinary catheter indications and CAUTI prevention was generally moderate to high, with more than half of the participants classified as having high overall knowledge. Most respondents correctly identified fundamental prevention measures; however, important misconceptions persisted. Knowledge gaps were particularly evident regarding prophylactic antimicrobial administration after catheter insertion and routine bladder irrigation with antimicrobial or iodine solutions. The knowledge of correct catheter fixation was notably limited, suggesting that technical aspects of catheter maintenance may be underemphasized in training and routine practice.

Participants’ perceptions were largely supportive of CAUTI prevention. Nearly all respondents agreed that education plays a key role in prevention, and most recognized catheter dwell time as an important risk factor. Despite this, self-reported practices revealed a clear knowledge-practice gap. While adherence to insertion-related measures such as hand hygiene before and after catheter insertion was high, routine maintenance practices were substantially weaker. Hand hygiene before catheter handling and the maintenance of a continuously closed drainage system were reported less consistently. This discrepancy is clinically important, as maintenance-phase behaviors, particularly minimizing catheter manipulation and performing hand hygiene before catheter contact, are critical for preventing both extraluminal and intraluminal contamination. The CDC emphasizes that prolonged catheterization is the most significant risk factor for CAUTI and that a substantial proportion of hospital-acquired UTIs are catheter-associated, underscoring the importance of proper maintenance and timely removal practices [6].

The pattern in our findings, of generally good knowledge but inconsistent maintenance practice, aligns with the broader literature. A mixed-methods systematic review of HCWs’ knowledge, attitudes, and practices found that positive attitudes are common. Yet, adherence to recommended practices can be suboptimal, often due to workflow constraints, perceived norms, and environmental barriers [17]. Similarly, cross-sectional KAP studies conducted in diverse healthcare settings report variable knowledge and practice levels, with practice frequently lagging behind knowledge. For example, studies have shown that the availability of guidelines, structured education, and supportive unit-level factors is associated with improved practices, highlighting the importance of organizational context, alongside individual knowledge [18,19].

Our multivariable analyses further suggest that both professional experience and prevention-related perceptions are associated with key maintenance behaviors. Correct catheter fixation after insertion was independently associated with longer overall work experience and higher total knowledge scores, while the maintenance of a continuously closed drainage system was associated with experience, profession, and perceptions regarding morbidity reduction. These findings align with implementation research indicating that adherence is shaped not only by knowledge but also by behavioral and contextual determinants, including local norms, leadership support, role clarity, and environmental cues [20].

Only a minority of participants reported receiving CAUTI-specific training in the preceding year, and recent training was associated with slightly higher prevention knowledge. This supports the value of ongoing, targeted educational refreshers, particularly focused on maintenance “micro-practices” such as hand hygiene before catheter manipulation, drainage system integrity, fixation techniques, and timely removal. However, education alone is unlikely to be sufficient. Evidence suggests that multicomponent interventions, such as reminders, stop orders, nurse-driven protocols, and bundled approaches, are more effective in reducing catheter utilization and CAUTI rates by decreasing device days [21,22]. Targeted system-level supports may therefore be especially beneficial in our setting, including standardized securement protocols, readily available fixation supplies, daily catheter necessity checklists, and documentation prompts for indication and planned removal. Notably, engineering and technology-based solutions may complement behavioral interventions, as demonstrated by evidence showing reduced catheter movement and lower CAUTI incidence with dedicated securement devices compared with adhesive tape [23].

Overall, our findings indicate that future improvement efforts should prioritize maintenance-phase behaviors, particularly hand hygiene before catheter handling, the preservation of closed drainage systems, and the minimization of unnecessary manipulation. These efforts should be supported by clear protocols for the management of unavoidable system breaks, standardized supplies, structured documentation, and an emphasis on timely catheter removal.

There are some limitations that should be considered. First, practices were self-reported and may therefore be subject to recall and social desirability bias, potentially leading to the overestimation of adherence to recommended infection prevention practices. Second, the cross-sectional design limits the ability to draw causal inferences. Third, the study was conducted in a single center, which may limit the generalizability of the findings. In addition, the questionnaire was developed based on current guidelines and relevant literature to capture clinically meaningful aspects of CAUTI prevention. The relatively low KR-20 values indicate the limited internal consistency of the knowledge scales. This may reflect the multidimensional nature of the items, as well as the high proportion of correct responses in several questions, suggesting limited variability and reduced ability to discriminate between different levels of knowledge. In addition, although content and face validity were assessed during questionnaire development, other aspects of measurement properties, such as test-retest reliability, were not evaluated and warrant further investigation in future studies. Therefore, the knowledge scores should be interpreted with caution. Finally, due to the anonymous design of the survey, information on non-responders was not available, and the potential for non-response bias cannot be excluded.

## Conclusions

Healthcare workers demonstrated generally adequate knowledge and positive attitudes toward CAUTI prevention. However, important gaps were identified in the consistent implementation of maintenance-related practices. These findings suggest that improving CAUTI prevention requires not only knowledge-based education but also the reinforcement of routine clinical behaviors and supportive organizational strategies.

Given the study’s limitations, future multicenter longitudinal studies using validated tools and objective measures of practice would help to confirm these findings and better inform targeted interventions.

## Appendices

Table 11 shows the questionnaire used. 

**Table 11:** Questionnaire: knowledge, perceptions, and practices of healthcare professionals regarding catheter-associated urinary tract infections (CAUTIs)

Part A. Demographic characteristics	
Q1. Gender	Male/female
Q2. Age (years)	___________
Q3. Educational level	Public or private school/bachelor’s degree in nursing/master’s degree/doctoral degree (PhD)/postdoctoral qualification/others (please specify): __________
Q4. Professional role	Nursing assistant/registered nurse/medical resident/medical specialist
Q5. Current department	Intensive care unit (ICU)/high-dependency unit (HDU)/internal medicine department/surgical department/oncology department/others (please specify): __________
Q6. Years of work experience in the current department	__________
Q7. Total years of professional experience	____________
Q8. Have you ever received additional training related to infections in general?	Yes/no
Q9. Did you have previous education in CAUTI in the last 12 months?	Yes/no
Part B. Knowledge assessment	
A. Knowledge of indications for urinary catheterization. Please indicate whether urinary catheterization is indicated in the following situations	
Q1. Narrowing of the urethra obstructing urine flow	Indicated/not indicated/I do not know
Q2. Neurogenic bladder due to paraplegia or tetraplegia	Indicated/not indicated/I do not know
Q3. Volume monitoring of urine output in an ambulatory patient without a pathological problem from the urinary tract	Indicated/not indicated/I do not know
Q4. Collection of urine samples for culture and antibiotic susceptibility testing of microorganisms (C&S) in ambulatory patients	Indicated/not indicated/I do not know
Q5. In patients who are expected to receive a large volume of intravenous fluids or diuretics during surgery	Indicated/not indicated/I do not know
Q6. In patients with prolonged immobilization	Indicated/not indicated/I do not know
Q7. To aid the healing of pressure ulcers in female incontinent patients	Indicated/not indicated/I do not know
Q8. In the context of palliative care in a terminal patient	Indicated/not indicated/I do not know
Q9. As a substitute for nursing care in patients with incontinence	Indicated/not indicated/I do not know
Q10. Before any kind of surgery as a routine procedure	Indicated/not indicated/I do not know
B. Knowledge of preventive measures for catheter-associated urinary tract infections (CAUTIs)	
Q1. Hand hygiene should be applied immediately before and after any handling of the urinary catheter	Yes/no/I do not know
Q2. An appropriately sized urinary catheter should be used	Yes/no/I do not know
Q3. The urinary catheter should be inserted only when necessary and removed as soon as possible to prevent urinary tract infection associated with urinary catheter use	Yes/no/I do not know
Q4. Other methods of urinary drainage should be considered before urinary catheter placement	Yes/no/I do not know
Q5. The urinary catheter should only be inserted by personnel trained in urinary catheter insertion under aseptic conditions	Yes/no/I do not know
Q6. Crushing of the urinary catheter should be avoided to keep the flow of urine unobstructed	Yes/no/I do not know
Q7. Flushing the bladder with an antimicrobial or iodine solution at least once a day will prevent urinary tract infection associated with urinary catheter use	Yes/no/I do not know
Q8. The urine collector should be emptied regularly to avoid the overflow of urine	Yes/no/I do not know
Q9. The urinary collector should always be kept below the level of the bladder	Yes/no/I do not know
Q10. The urine collector should not rest on the floor	Yes/no/I do not know
Q11. Prophylactic antimicrobial treatment should be given after the insertion of the urinary catheter	Yes/no/I do not know
Q12. The urinary catheter should be removed as soon as the patient does not need it	Yes/no/I do not know
Q13. The urinary catheter should be clipped on the inner side of the thigh in women or on the lower abdomen in men	Yes/no/I do not know
Q14. Sterile lubricants should be used when inserting the urinary catheter to reduce the irritation of the urethra	Yes/no/I do not know
Q15. The perineal area in women should be cleaned from back to front	Yes/no/I do not know
Part C. Perceptions regarding catheter-associated urinary tract infections	
Q1. Urinary tract infection associated with urinary catheter use is a serious disease that can lead to death	Strongly disagree/disagree/neither agree nor disagree/agree/strongly agree
Q2. Changing a urinary catheter in routine is a practice that helps prevent urinary tract infections associated with urinary catheter use	Strongly disagree/disagree/neither agree nor disagree/agree/strongly agree
Q3. The occurrence of urinary tract infection associated with urinary catheter use cannot be avoided in patients who have undergone bladder catheterization	Strongly disagree/disagree/neither agree nor disagree/agree/strongly agree
Q4. Education on the basics of urinary catheter care helps prevent urinary tract infections associated with urinary catheter use	Strongly disagree/disagree/neither agree nor disagree/agree/strongly agree
Q5. Healthcare professionals can remove the catheter whenever it is convenient for them	Strongly disagree/disagree/neither agree nor disagree/agree/strongly agree
Q6. Urinary tract infections associated with urinary catheter use are a common problem	Strongly disagree/disagree/neither agree nor disagree/agree/strongly agree
Q7. Aseptic technique required for the removal of the Foley urinary catheter	Strongly disagree/disagree/neither agree nor disagree/agree/strongly agree
Q8. Workload affects my ability to implement the guidelines for the prevention of CAUTI	Strongly disagree/disagree/neither agree nor disagree/agree/strongly agree
Q9. The proper placement and maintenance of the urinary catheter can reduce the risk of CAUTI-related morbidity	Strongly disagree/disagree/neither agree nor disagree/agree/strongly agree
Q10. The proper placement and maintenance of the urinary catheter can reduce the risk of CAUTI-related mortality	Strongly disagree/disagree/neither agree nor disagree/agree/strongly agree
Q11. In the event that I observed improper practices being followed during urinary catheter insertion, I would intervene and politely point out the error	Strongly disagree/disagree/neither agree nor disagree/agree/strongly agree
Q12. Urinary catheter dwell time contributes significantly to the occurrence of CAUTI	Strongly disagree/disagree/neither agree nor disagree/agree/strongly agree
Part D. Practices related to catheter-associated urinary tract infections	
Q1. When do you perform hand hygiene? (Select all that apply)	
Q1.1. Before and after urinary catheter insertion	
Q1.2. Before handling the urinary catheter	
Q1.3 After urinary catheter insertion	
Q2. Which of the following do you use/adhere to during urinary catheter insertion? (Select all that apply)	
Q2.1. Sterile equipment (e.g., sterile gloves and sterile gauze)	
Q2.2. Aseptic technique	
Q2.3. Cleaning the urethral meatus with an antiseptic solution	
Q2.4. Ensuring appropriate catheter stabilization after insertion	
Q2.5. Single-use lubricating gel	
Q3. Which of the following do you use/adhere to during routine catheter care? (Select all that apply)	
Q3.1. Keeping the urinary catheter and urinary collector tube free of folds for unobstructed flow of urine	
Q3.2. Keeping the urinary tract at a level lower than the bladder	
Q3.3. Emptying the urine collector regularly and using a different urine collection container for each patient	
Q3.4. Keeping the system continuously closed	
Q3.5. Use of appropriate personal protective equipment (PPE) according to the guidelines when handling the urinary catheter	
Q4. Which of the following do you use/adhere to during urinary catheter removal? (Select all that apply)	
Q4.1. Use of PPE, due to the risk of exposure to biological fluids	
Q4.2. Use a 10 or 20 mL syringe to remove water from the urinary catheter stabilization balloon	
Q4.3. Recording the date of the removal of the urinary catheter	
Q4.4. Use of the appropriate technique to remove the fixation medium	
